# Clinical experience with biologic treatment in resistant eosinophilic fasciitis: Case reports and review of the literature

**DOI:** 10.1097/MD.0000000000025359

**Published:** 2021-04-02

**Authors:** Daniel Erez, Yehuda Shoenfeld, Ayman Natour, Zamir Dovrish, Oshrat E. Tayer-Shifman, Yair Levy

**Affiliations:** a Department of Medicine D, Meir Medical Center, Kfar Saba; b Sackler Faculty of Medicine, Tel Aviv University, Tel Aviv; c St. Petersburg State University, St. Petersburg, Russia; d Zabludowicz Center for Autoimmune Diseases, Sheba Medical Center, Tel Hashomer; e Department of Medicine E, Meir Medical Center; f Rheumatology Service, Meir Medical Center, Kfar Saba, Israel.

**Keywords:** Eosinophilic fasciitis, IVIG, relapse, rituximab, steroid-refractory

## Abstract

**Rationale:**

Eosinophilic fasciitis (EF) is an uncommon connective tissue disorder characterized by limb and trunk erythema, with symmetrical thickening of the skin. Its pathogenesis is poorly understood. Treatment consists mainly of glucocorticoids. Yet, no randomized trials have evaluated therapies for this rare disease and the optimal treatment modality remains unclear. Although most patients show partial or complete response to glucocorticoids, many relapse upon drug tapering, while others either do not respond at all or fail to sustain prolonged remission. Second-line therapy for this rare disorder includes mainly methotrexate (MTX), azathioprine, cyclosporine and hydroxychloroquine. Recently, several attempts using rituximab and intravenous immunoglobulins (IVIG) have shown good clinical results.

**Patient concerns:**

The three patients had good clinical response to glucocorticoid treatment, followed by disease flare when the drug dose was tapered. Adding methotrexate in all patients and azathioprine to patient 3 did not lead to remission.

**Diagnoses:**

EF was diagnosed in all patients based on clinical presentation accompanied by fascia biopsy that demonstrated eosinophilic fasciitis.

**Interventions:**

The patients were successfully treated with rituximab or IVIG, achieving sustained remission.

**Outcomes:**

The three cases had good clinical response to glucocorticoid treatment, followed by disease flare when the drug dose was tapered. The patients were then successfully treated with rituximab or IVIG, achieving sustained remission.

**Lessons:**

This review of three cases of EF supports the results of previous reports, suggesting addition of rituximab and IVIG is an effective treatment for patients with refractory disease.

## 1 Introduction

Eosinophilic fasciitis (EF), first described by Shulman in 1975, is a rare disorder of unknown etiology and poorly understood pathogenesis.^[1]^ Since then, about 300 cases have been reported.^[2–5]^ It is characterized by an early phase of limb or trunk erythema and edema, which later progresses to collagenous thickening of the subcutaneous fascia. Suggested triggers to EF development include certain medications (phenytoin, Ramipril and subcutaneous heparin),^[6]^ graft-versus-host-disease, autoimmune diseases (systemic lupus erythematosus, Sjogren's syndrome, and primary biliary cirrhosis)^[7–9]^ and hematologic disorders. Almost all patients have symmetrical skin involvement. The temporal evolution includes non-pitting edema followed by induration with puckering that has the texture of orange peel (peau d’orange). The initial edematous phase may be indistinguishable from early sclerodermatous skin changes. Yet, sclerodactyly and Raynaud's phenomenon are usually absent, and the skin of the hands and feet is generally spared. While most patients with EF have peripheral blood eosinophilia,^[4]^ it is generally transient and does not correlate with disease severity.^[2]^ Over 50% of patients have elevated C-reactive protein (CRP) and polyclonal hypergammaglobulinemia.^[4,10]^

No randomized trials have evaluated therapies for EF; thus, the best approach remains unclear. The mainstay of treatment includes systemic glucocorticoids, starting at doses equivalent to 1 mg/kg per day of prednisone.^[4,5,11]^ There is often rapid resolution of peripheral eosinophilia and normalization of erythrocyte sedimentation rate (ESR), and affected skin gradually softens. However, many patients do not achieve complete response.^[4]^ Additionally, relapses may occur. Immunosuppressive drugs (ISD), such as azathioprine, cyclosporine, methotrexate (MTX) and rituximab are mostly initiated due to failure or dependence on high doses of corticosteroids.^[4,5,12–17]^ Here we present a series of three patients who presented to the Rheumatology Clinic at Meir Medical Center with a diagnosis of eosinophilic fasciitis during 2002–2007. We followed their clinical course according to various treatment modalities.

## 2 Ethical review

This study was approved by the Meir Medical Center Institutional Review Board for clinical research. Approval number 0297-19-MMC. Approval date 10.11.2019.

## 3 Case reports

### 3.1 Patient 1

In September 2002, a 56-year-old man with hypertension described progressive swelling and induration of upper and lower extremities for the past month. In addition, he described progressive weakness and joint pain, without fever. Creatinine kinase (CK) was within normal values. Physical examination revealed stiff, rock-hard skin of upper and lower limbs, with sparing of the hands and feet. Raynaud phenomenon was absent. Laboratory values revealed elevated CRP of 7 mg/dl (normal <0.5 mg/dl) and significant eosinophilia of 4570/mm^3^ (normal 300–500/mm^3^). Quantitative immunoglobulin tests showed elevated immunoglobulin G of 2250 mg/dl (normal 900–1500 mg/dl). Antinuclear antibody (ANA) and complement levels were normal. Deep skin biopsy from the right upper limb showed chronic inflammation with numerous eosinophils. The inflammatory infiltrate involved predominantly the septa and partially the skeletal muscle. The findings were consistent with eosinophilic fasciitis. Prednisone was initiated at a dose of 1 mg/kg. The patient was treated with a 4-month course with gradual tapering down. With this regimen, he experienced improvement in skin manifestations and the eosinophil count normalized. However, during drug tapering the patient quickly relapsed. He consequently received MTX as a steroid sparing agent for 2 weeks, during which time he developed drug-related nausea and vomiting. As a next step, the patient was treated with high-dose intravenous immune globulin (IVIG) at a dose of 2 g/kg/month from 2003–2015, after which the treatment was stopped, with no further relapses. With the above regimen, the patient demonstrated clinical stability, except for one exacerbation in 2008, which was accompanied by a rise in eosinophil count to 1300/mm^3^, CRP of 1.7 mg/dl and immunoglobulin G 3680 mg/dl. He regained remission on IVIG treatment and did not experience a relapse until his death from unrelated causes, in 2018.

### 3.2 Patient 2

In November 2007, a 61-year-old woman with no significant background diseases presented with a 2-month history of myalgia, accompanied by swelling of the face, and upper and lower limbs. She also reported muscle cramps, initially in her legs that gradually progressed to the arms and neck. Additional clinical features were weakness, palpitations and weight loss of 5 kg during that period. On physical examination, there was marked thickness and stiffness of the skin of both upper and lower extremities. Laboratory evaluation revealed elevated CRP of 6.73 mg/dl, hypoalbuminemia of 2.6 g/dl (normal 3.5–5.20 g/dl), moderate eosinophilia of 1800/mm^3^, with normal immunoglobulin levels. Whole body computed tomography (CT) was without significant findings. Echocardiography and continuous ECG (Holter) monitoring were normal. A bone marrow biopsy was negative for malignancy or other pathology. Gallium scan did not demonstrate major pathology. Stool examination was negative for parasites. Gastroscopy with biopsies did not demonstrate pathologic changes apart from mild gastritis. Nevertheless, a suspicion of *Trichinella spiralis* was raised and the patient was treated with albendazole. However, there was only partial clinical improvement, as peripheral eosinophilia count did not regress and CRP levels continued to rise. Moreover, serology for *T. spiralis* was negative. The patient underwent deep skin and muscle biopsies in the left leg, which were consistent with eosinophilic fasciitis.

Prednisone was initiated at a dose of 60 mg daily, with rapid resolution of peripheral eosinophilia and hypoalbuminemia and decrease in CRP to normal levels. However, the patient became steroid dependent, with rapid relapse of her symptoms upon drug tapering. She also developed marked hyperglycemia. A trial of MTX was not tolerated due to nausea. Azathioprine was initiated at a dose of 50 mg three times a day, along with continuation of low dose steroids. She continued with this treatment for additional 2 years (2008–2010). She then continued low dose prednisone (10–20 mg) until October 2017, when skin induration and swelling recurred, accompanied by positive groove sign. CRP levels increased mildly to 0.7 mg/dl, with no peripheral eosinophilia. At this stage, the patient received induction treatment with rituximab, at a dose similar to the regimen used for vasculitis (1 g rituximab, followed by a similar dose 2 weeks later). She then continued maintenance therapy with a similar course of rituximab 6 months later, along with prednisone at a maintenance dose of 5 mg per day. With this treatment regimen, the skin changes regressed dramatically. The patient regained complete remission and has not experienced a relapse.

### 3.3 Patient 3

A 57-year-old woman with a history of hypertension, hyperlipidemia and ischemic heart disease presented in June 2006 due to progressive skin thickening and induration for the previous 3 months, mostly in the hands, face and upper back. Accompanying symptoms were fatigue, burning sensation in the hands and inability to take deep breaths. Raynaud phenomenon was absent. Skin examination revealed a woody texture compatible with peau d’orange. Moderate hypoalbuminemia was present, with mild peripheral eosinophilia of 1000/mm^3^. Hypergammaglobulinemia was not present, and CRP levels were mildly elevated at 1 mg/dl. Deep skin biopsy from the left arm demonstrated marked collagen thickening, accompanied by rich perivascular lymphohistiocytic infiltrates, compatible with eosinophilic fasciitis. The patient began treatment with high dose prednisone plus MTX, with rapid improvement. Nevertheless, her skin manifestations soon recurred when steroid dose was gradually reduced. In October 2006, intravenous cyclophosphamide was initiated along with glucocorticoids; 6 doses of 500 mg at 2-week intervals, with no major improvement. Next, she began IVIG treatment at a dose of 2 g/kg in monthly intervals during 2007–2011 with low dose glucocorticoids. With this treatment, she regained clinical remission until April 2011, when skin induration of the arms and upper back worsened. CRP was elevated at 4.28 mg/dl, without peripheral eosinophilia. At this point, the patient was given rituximab at a dose of 1 g, followed by a similar dose two weeks later. With the above treatment, accompanied by prednisone at doses of 5–10 mg, she regained clinical remission, with no further relapses.

## 4 Discussion

Eosinophilic fasciitis is an extremely uncommon connective tissue disease, first described by Shulman in 1975.^[2]^ Its hallmark is symmetric swelling, induration and thickening of the skin and subcutaneous tissue of distal extremities. Skin changes evolve through three stages. Most patients appear to present with an edematoid phase, followed by peau d’orange appearance of the skin. Finally, tight skin and induration predominate. However, various stages may be present simultaneously. Localized morphea has been described in up to 30% of cases.^[4]^

The disease is slightly more common in males than females and can occur at any age.^[18]^ Though rare, its clinical manifestations can mimic other more common diseases, such as scleroderma. This raises the possibility that the true prevalence is higher and many patients are misdiagnosed. Nevertheless, while clinical and pathological features are well defined, therapeutic management remains a great challenge.

The mainstay of therapy rests on glucocorticoids at a dose of 0.5–1 mg/kg/d. However, patients may take up to 4 years to recover and relapse rate is high. Partial or complete response is seen in just over 60% of patients, as described by Lakhanpal et al ^[4]^ Moreover, prolonged courses of high dose glucocorticoids predispose patients to glucocorticoid-induced osteoporosis and opportunistic infections.

Lebeaux et al ^[11]^ described a series of 32 patients with EF, all treated with oral glucocorticoids as first-line therapy. Among them, 44% required ISD in addition to corticosteroids, due to unsatisfactory response. The main immunosuppressive drug used in their study was MTX, which retrospectively, was found to be used more frequently in patients with morphea-like lesions. In addition, methylprednisolone pulses at the beginning of therapy were associated with higher complete remission rates. It was also shown that delay in diagnosis of more than 6 months was associated with decreased likelihood of complete remission.

Interestingly, morphea-like lesions were also significantly associated with residual fibrosis in systematic review of the literature.^[3]^ The 3 patients in our study did not have morphea-like lesions and were all diagnosed after 3 to 4 months of symptoms. Yet, none received pulse methylprednisolone therapy when treatment was initiated.

As mentioned above, ISD are commonly used in cases of steroid-dependent disease, for steroid-resistant patients and for patients who relapse during follow-up. Drugs commonly described in previous reports included MTX, azathioprine, hydroxychloroquine and cyclosporine.^[4,12,19]^

Lakhanpal et al ^[4]^ described favorable outcomes using a combination of corticosteroid and hydroxychloroquine. In another report of 12 cases,^[5]^ other medications in addition to corticosteroids included hydroxychloroquine, topical tacrolimus, MTX, sulfasalazine and cyclosporine.

However, there have been anecdotal case reports of success with intravenous immunoglobulins used to treat refractory eosinophilic fasciitis.^[20,21]^ Pimenta et al ^[22]^ described a case of a 39-year-old man who was admitted for swelling and induration of the hands and feet, accompanied by severe functional impairment. Laboratory findings included peripheral eosinophilia of >3000/mm^3^ and CRP 17.8 mg/L. IgG levels were elevated at 2000 mg/dl (normal range 650–1500). Biopsy findings from skin, deep fascia and muscle were compatible with eosinophilic fasciitis. Oral prednisone was initiated at a dose of 30 mg/d. After 2 months, minimal clinical improvement was apparent, although peripheral eosinophilia normalized and CRP decreased to 1.6 mg/L. Gradual prednisone tapering to less than 20 mg/d resulted in clinical flare. Addition of methotrexate for 7  months resulted in only slight clinical improvement. IVIG treatment was given at a dose of 0.5 mg/kg/d on 3 consecutive days followed by 5 additional treatments at 1-month intervals. He then regained remission, apart from mild cutaneous induration over the legs. Two years after discontinuing IVIG, he was clinically stable on 2.5 mg prednisone/day and 10 mg methotrexate/week, with normal laboratory test results.

Rituximab, a chimeric monoclonal antibody, targets the CD-20 antigen present on the surface of B-lymphocytes. In a previous report, it was used successfully for the treatment of a patient with eosinophilic fasciitis and hypergammaglobulinemia,^[16]^ and in a patient with EF associated with aplastic anemia.^[23]^ Nahhas et al ^[24]^ described a 67-year-old male presenting with a 3-month history of diffuse myalgia and skin induration and swelling. All extremities were firm to palpation, with positive groove sign in both arms. Biopsy and EMG findings were consistent with EF. Treatment with oral prednisone, pulse methylprednisolone, mycophenolate mofetil, cyclophosphamide and IVIG were without marked clinical improvement. Following combination treatment of prednisone, methotrexate and rituximab (1 g IV given 2 weeks apart, followed by another course 5 months later, skin swelling and tautness improved markedly and peripheral eosinophilia normalized.

The three patients described in this series all presented with steroid-sensitive disease. However, all three experienced disease flare as prednisone was tapered down; hence, they were steroid dependent. Almost half of patients with EF require additional immunosuppressive agents due to inadequate steroid response or disease flare with drug tapering. As described by Lebeaux et al,^[11]^ the most frequently used immunosuppressive drug was MTX, which was also the first additional drug of this type given in our series. However, two of the patients experienced adverse effects which necessitated its discontinuation. Other ISD described in our series either failed to achieve remission (cyclophosphamide) or could not sustain remission.

Interestingly, prolonged IVIG treatment (for 7 years and 4 years in cases 1 and 3, respectively), at a maintenance dose of 2 g/kg monthly, succeeded in maintaining clinical remission with complete resolution of skin swelling and induration. In the report by Pimenta et al,^[22]^ similarly to the cases presented in our review, MTX was used as the first additional immunosuppressive drug. IVIG treatment was commenced following inadequate clinical response. As opposed to the treatment regimen given to our patients, which included higher doses of IVIG for a longer treatment course, a dose of 0.5 mg/kg/d with maintenance doses up to 5  months induced remission, which was sustained 2 years following treatment cessation. It is important to note that the patients receiving IVIG in our study were older (56 and 57 years for cases 1 and 3, respectively, as compared to 39 years in the cited report), which could influence treatment response. Gamma globulins were elevated in case 1, similarly to the case presented by Pimenta, whereas case 3 had normal immunoglobulin levels. Interestingly, as noted above, case 1 maintained remission for a longer period than case 3 did and did not require additional treatment, while case 3 experienced a major relapse and was later treated with rituximab. Thus, hypergammaglobulinemia could, by some means, play a role in treatment response to IVIG. In addition, our cases continued IVIG therapy indefinitely and except for one disease flare, maintained remission for prolonged periods (7 and 4 years for cases 1 and 3, respectively), while the case presented by Pimenta received a relatively short course of 6 cycles, after which he maintained remission. However, last follow-up was 2 years after treatment discontinuation. This calls into question whether patients with EF should be maintained on IVIG therapy for prolonged periods.

Hypergammaglobulinemia was also observed in a patient with EF who was successfully treated with rituximab.^[16]^ Cases 2 and 3 in our report were treated with rituximab and did not experience hypergammaglobulinemia. Nevertheless, good clinical response was observed in both, on a regimen similar to that described by Nahhas et al ^[24]^ The rapid response to rituximab observed in our cases and in those cited above, suggests an anti-CD20 mechanism may be involved in treatment response. The cases discussed above are summarized in Table 1.

**Table 1:** Summary of cases according to biologic treatment and previous therapy.

Case	Sex	Age	Previous treatment	Biologic treatment	Treatment regimen	Response	Relapse and treatment
Scheinberg 2006^[16]^	Female	20	Not mentioned	Rituximab	Unknown	CR	No
Pimenta 2009 ^[22]^	Male	39	Prednisone, MTX	IVIG	0.5 g/kg/d for 3 consecutive days (6 courses)	CR	No
de Masson 2013^[23]^	Male	57	Prednisone, cyclosporine	Rituximab	375 mg/m^2^/week x 4 weeks	CR	Yes, Rituximab
Nahhas 2014^[24]^	Male	67	Prednisone, MTX, mycophenolate mofetil, hydroxychloroquine, cyclophosphamide	IVIG, rituximab	Not mentioned 1 g (2 doses 2 weeks apart), 1 g following 6 months	CR	No
Case 1	Male	56	Prednisone, MTX	IVIG	2 g/kg monthly	CR	Yes, IVIG
Case 2	Female	61	Prednisone, MTX, azathioprine	Rituximab	1 g (2 doses 2 weeks apart), 1 g after 6 months	CR	No
Case 3	Female	57	Prednisone, MTX, cyclophosphamide	IVIG	2g/kg monthly, 2007–2011	CR	Yes, Rituximab

CR = complete remission, IVIG = intravenous immunoglobulin, MTX = methotrexate.

None of our patients received pulse methylprednisolone at the beginning of treatment. This could be associated with poor outcomes.^[11]^ None of the patients in our series presented with clinical or histological morphea-like lesions, which have also been associated with poor outcomes.^[3,11]^

In conclusion, this study presents clinical and laboratory information of 3 patients with EF. Due to the small number of patients and the rarity of case descriptions in previous reports, it is difficult to draw conclusions regarding the preferred treatment regimen for the disease. However, as described in our study and in the current literature, steroid refractory disease is a major problem in EF. Our findings support the notion that adding intravenous immunoglobulins and rituximab to the armamentarium for treatment-resistant cases could prove useful in managing this rare disease.

## Acknowledgments

The authors would like to acknowledge Faye Schreiber, MS who edited the manuscript. She is an employee of Meir Medical Center.

## Author contributions

**Conceptualization:** Daniel Erez, Yair Levy.

**Data curation:** Daniel Erez, Ayman Natour, Zamir Dovrish.

**Investigation:** Ayman Natour, Zamir Dovrish.

**Writing – original draft:** Daniel Erez, Ayman Natour, Zamir Dovrish, Yair Levy.

**Writing – review & editing:** Daniel Erez, Yehuda Shoenfeld, Oshrat E. Tayer-Shifman, Yair Levy.
